# Endoglin/CD105-Based Imaging of Cancer and Cardiovascular Diseases: A Systematic Review

**DOI:** 10.3390/ijms22094804

**Published:** 2021-04-30

**Authors:** Vincent Q. Sier, Joost R. van der Vorst, Paul H. A. Quax, Margreet R. de Vries, Elham Zonoobi, Alexander L. Vahrmeijer, Ilona A. Dekkers, Lioe-Fee de Geus-Oei, Anke M. Smits, Weibo Cai, Cornelis F. M. Sier, Marie José T. H. Goumans, Lukas J. A. C. Hawinkels

**Affiliations:** 1 Department of Surgery, Leiden University Medical Center, 2300 RC Leiden, The Netherlands; v.q.sier@lumc.nl (V.Q.S.); j.r.van_der_vorst@lumc.nl (J.R.v.d.V.); p.h.a.quax@lumc.nl (P.H.A.Q.); m.r.de_vries@lumc.nl (M.R.d.V.); e.zonoobi@lumc.nl (E.Z.); a.l.vahrmeijer@lumc.nl (A.L.V.); 2 Edinburgh Molecular Imaging Ltd. (EMI), Edinburgh EH16 4UX, UK; 3 Department of Radiology, Leiden University Medical Center, 2300 RC Leiden, The Netherlands; i.a.dekkers@lumc.nl; 4 Department of Radiology, Section of Nuclear Medicine, Leiden University Medical Center, 2300 RC Leiden, The Netherlands; l.f.de_geus-oei@lumc.nl; 5 Biomedical Photonic Imaging Group, University of Twente, 7500 AE Enschede, The Netherlands; 6 Department of Cell & Chemical Biology, Leiden University Medical Center, 2300 RC Leiden, The Netherlands; a.m.smits@lumc.nl (A.M.S.); m.j.t.h.goumans@lumc.nl (M.J.T.H.G.); 7 Departments of Radiology and Medical Physics, University of Wisconsin-Madison, Madison, WI 53705, USA; wcai@uwhealth.org; 8 Percuros B.V., 2333 CL Leiden, The Netherlands; 9 Department of Gastroenterology and Hepatology, Leiden University Medical Center, 2300 RC Leiden, The Netherlands; l.j.a.c.hawinkels@lumc.nl

**Keywords:** endoglin, CD105, TGF-β, imaging, image-guided surgery, molecular imaging, nanoparticles, microbubbles, angiogenesis, cancer, cardiovascular diseases

## Abstract

Molecular imaging of pathologic lesions can improve efficient detection of cancer and cardiovascular diseases. A shared pathophysiological feature is angiogenesis, the formation of new blood vessels. Endoglin (CD105) is a coreceptor for ligands of the Transforming Growth Factor-β (TGF-β) family and is highly expressed on angiogenic endothelial cells. Therefore, endoglin-based imaging has been explored to visualize lesions of the aforementioned diseases. This systematic review highlights the progress in endoglin-based imaging of cancer, atherosclerosis, myocardial infarction, and aortic aneurysm, focusing on positron emission tomography (PET), single-photon emission computed tomography (SPECT), magnetic resonance imaging (MRI), near-infrared fluorescence (NIRF) imaging, and ultrasound imaging. PubMed was searched combining the following subjects and their respective synonyms or relevant subterms: “Endoglin”, “Imaging/Image-guided surgery”. In total, 59 papers were found eligible to be included: 58 reporting about preclinical animal or in vitro models and one ex vivo study in human organs. In addition to exact data extraction of imaging modality type, tumor or cardiovascular disease model, and tracer (class), outcomes were described via a narrative synthesis. Collectively, the data identify endoglin as a suitable target for intraoperative and diagnostic imaging of the neovasculature in tumors, whereas for cardiovascular diseases, the evidence remains scarce but promising.

## 1. Introduction

In the fields of oncology and cardiovascular disease, imaging of pathologic lesions is essential for diagnosis, staging, evaluation of drug effectiveness, and follow-up. Clinical imaging techniques currently rely heavily on pre- and post-operative imaging, visualizing pathologies and pathophysiological processes after resection (e.g., immunohistochemistry) or during check-ups (e.g., computed tomography (CT)-scan). For example, the commonly used microvessel density (MVD) evaluation involves taking a biopsy, followed by immunohistochemistry for endothelial cell markers like CD31, CD34, CD105 and assessment by a pathologist. Even though MVD is a prognostic marker for various types of cancer (e.g., gastrointestinal tumors, malignant melanomas, and tumors of the central nervous system), real-time imaging modalities focusing on, in principle, the same molecular targets offer a broader potential [1,2,3,4]. Clinical translation of this concept into a theragnostic as well as intraoperative setting is strongly supported by the wide availability of clinically used radionuclide, paramagnetic, fluorescent, or gas-filled tracers, visualized by techniques such as single positron emission tomography (SPECT), positron emission tomography (PET), magnetic resonance imaging (MRI), near-infrared fluorescence (NIRF) imaging, and contrast-enhanced ultrasound (CEUS) imaging. With regard to intraoperative applications, NIRF imaging is one of the most suitable techniques. While relying on the same principle of molecular targeting as (in vivo) PET, SPECT and (in vitro) immunohistochemical analysis, NIRF imaging can be used in the context of image-guided surgery (IGS), allowing surgeons to discern between healthy and malignant tissue [5]. The selection of appropriate targets is of key importance to clinical imaging and related research. The previously mentioned endothelial cell marker CD105, better known as endoglin, is an interesting candidate target to visualize various diseases.

**Figure 1:**
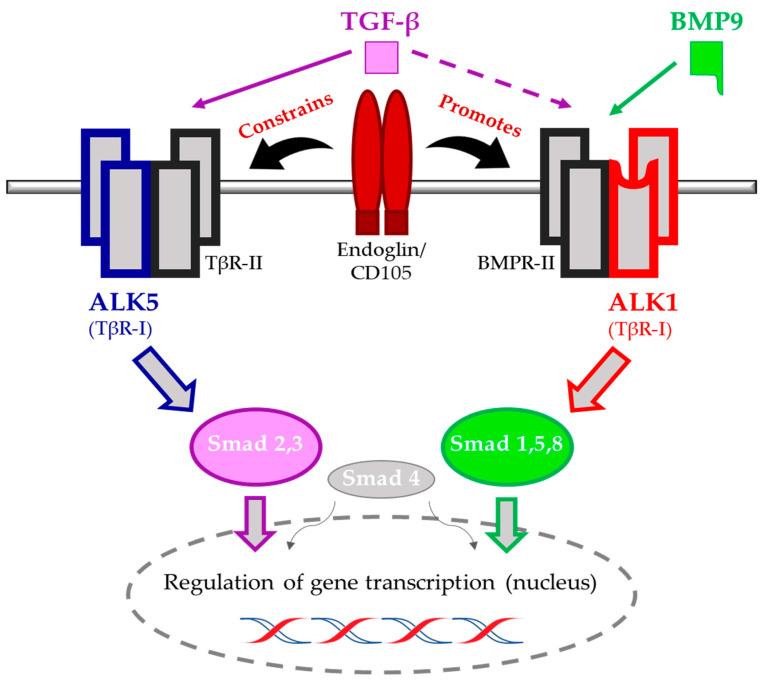
Overview of the two dominant TGF-β signaling pathways in endothelial cells. Presence of endoglin/CD105 on the cell membrane upon binding of TGF-β is associated with promotion of the angiogenesis-favoring ALK1 pathway, while constraining the mainly antagonistically functioning ALK5 pathway. The ALK1 pathway can also be initiated separately via BMP9 (and BMP10 during embryogenesis).

Endoglin is a 658 amino acid long, homodimeric transmembrane glycoprotein of 180 kDa [6,7]. Used as a marker for activated endothelial cells, endoglin has proven itself as a potent prognostic indicator for cancer patients, predicting poor outcomes for overall, disease free, and cancer-specific survival [8]. However, endoglin is rapidly surpassing its label of mere endothelial cell activation marker. As a coreceptor for transforming growth factor (TGF)-β1 and -β3, endoglin interacts with TGF-β type I and II receptors (TβR-I, TβR-II). In addition, it modulates bone morphogenetic protein (BMP)9/10 signaling (Figure 1) [9,10]. Active TGF-β binds to TβR-II, leading to recruitment and phosphorylation of TβR-I, also known as activin receptor-like kinase (ALK)5. Subsequently, the activated TβR-I kinase phosphorylates receptor-regulated SMADs, SMAD2/3 (R-SMADs). Phosphorylated R-SMADs bind to the co-SMAD, SMAD4. They migrate to the nucleus to regulate gene transcription [11,12]. In endothelial cells, TGF-β can signal via two type I receptors: ALK5 and ALK1, via, respectively, inducing SMAD2/3 and SMAD1/5/8 phosphorylation. Endoglin has multiple roles in this signaling cascade. While ALK1 and TβRII form a complex with endoglin on the cell surface, association of ALK5 with endoglin is dependent on the binding of TGF-β1 to TβRII and recruitment of ALK1 to the receptor complex [13,14]. Additionally, BMP9 and BMP10 can bind to endoglin, ALK1, and a bone morphogenetic protein type II receptor (BMPR-II) and induce SMAD1/5/8 phosphorylation via ALK1-dependent mechanisms (Figure 1) [15].

Presence of endoglin on the cell membrane constrains the ALK5-signaling route, thereby stimulating the endothelial cell proliferation-favoring activity of ALK1. Accordingly, increased expression of endoglin has been mostly linked to activated endothelial cells at sites of inflammation and angiogenesis. Moreover, a complex of ALK1 and endoglin binds BMP9 directly, enabling the induction of proangiogenic processes [15].

Next to its key role in regulating angiogenesis, endoglin is expressed by neoplastic epithelial cells, hematopoietic stem cells, innate immune cells, adaptive immune cells, (cancer-associated) fibroblasts, and mesenchymal stem cells, as reviewed by Schoonderwoerd and colleagues [14]. These cells express endoglin mostly in the context of complex TGF-β-regulated pathologic lesions like cancer, cardiovascular diseases, and fibrosis, suggesting that endoglin may be employed to target various pathologic conditions for therapy or imaging [14,16]. The intricacy of endoglin-mediated mechanisms is illustrated by a specific subtype of Rendu–Osler–Weber disease: hereditary hemorrhagic telangiectasia type 1 (HHT1). This rare, autosomal dominantly inherited disease is characterized by mutations in the endoglin gene, resulting in pulmonal arteriovenous malformations and telangiectasia-mediated epistaxis [17]. Next to endothelial cell defects, HHT1 patients have an impaired immune system, characterized by lymphopenia of CD4+ (helper) T cells, CD8+ (cytotoxic) T cells, and natural killer cells, in addition to functional deficits in neutrophils, polymorphonuclear cells, and monocytes [18,19,20].

In cardiovascular research, there is a strong need for a noninvasive tool to study neovessel formation in a preclinical setting, in which researchers heavily rely on histologic analyses, and in a clinical setting, where the quantification of in vivo intralesional angiogenesis is not yet common. Although investigated to a lesser extent than HHT1 and cancer, the role of endoglin in atherosclerosis, specifically in endothelial cell activation, vascular remodeling, angiogenesis, and inflammatory cell migration, suggest that the protein is a versatile imaging target [21,22]. Of specific interest would be the visualization and quantification of angiogenic processes to assess and monitor disease driving mechanisms like intrawall neoangiogenesis and intraplaque hemorrhage. Such an additional layer to traditional imaging modalities would ideally be combined with the benefits of existing noninvasive, real-time techniques such as ultrasonic (three-dimensional) wall and lumen size determination and measurements of hemodynamic parameters.

For tumors, endoglin-based imaging may provide added value in disease monitoring, quantification, and therapy decision in a (pre) clinical setting. In vivo imaging of endoglin expression on neoangiogenic vasculature could become a noninvasive alternative to MVD determination, allowing for improved disease classification, disease monitoring, therapy decision making, and personalizing the choice of (anti-angiogenic) treatments. Moreover, live endoglin-based imaging can be employed to perform IGS in highly vascularized tumors, whether or not combined with multiple other targets and preoperative mapping using hybrid tracers.

This review will give a systematic overview of the use of endoglin as a molecular target for imaging of cancer and cardiovascular diseases, including (pre) clinical applications such as IGS and diagnostics. The aim is to identify the current status of endoglin-based imaging of pathologic lesions in a (pre) clinical setting for cancer and cardiovascular diseases. In the method section, the systematic method of literature search will be described. Section 3 will discuss the general function of endoglin in cancer, including its role in angiogenesis and other processes, and the rationale of using endoglin as target for tumor imaging. Next, the current literature on (pre) clinical, endoglin-based, PET, MR, NIRF, and ultrasound imaging in tumors will be reviewed, followed by the role of endoglin in imaging of vascular disorders. The last sections will summarize and discuss the possibilities and limitations of endoglin-based imaging, as well as consider future perspectives.

## 2. Methods

### 2.1. Search Strategy

An initial orientational search was performed in PubMed and Google Scholar to identify appropriate search terms for endoglin-based imaging. These terms were consequently used in an extensive literature search in PubMed: (i) endoglin and (ii) imaging and image-guided surgery. All relevant Mesh terms, appropriate search field tags, applicable synonyms, and specific subterms were added to the final search strategy, which was performed on 17 December 2020 (Appendix A). This systematic review was carried out following the Preferred Reporting Items for Systematic Reviews and Meta-Analyses (PRISMA) guidelines of 2009, and the protocol, based on the basic format by Systematic Review Center for Laboratory animal Experimentation (SYRCLE), has been made publicly available [23,24,25]. The authors of this manuscript are aware of the recently published updated PRISMA 2020 statement [26]. Our research has been designed and conducted under the PRISMA 2009 guidelines and is thus published accordingly.

### 2.2. Eligibility Criteria

The following inclusion criteria were employed: (i) published in the English language, (ii) making use of one of the following imaging modalities: PET, SPECT, MRI, NIRF, ultrasound, (iii) report of an imaging modality-specific outcome measure that should be capable of demonstrating both contrast differences and feasibility of endoglin-based imaging, (iv) experimental study using (parts/derivatives of) animals or human subjects for cancer- or cardiovascular disease-related research. The final decision of including ex vivo studies in human organs has been made afterwards, upon finding only one such a study in the definitive literature search. The following exclusion criterion was employed: articles that reported on soluble endoglin. All included articles had to meet every in- and exclusion criterion. Full-texts were available for each eligible study and did not influence in- or exclusion of articles. All eligible studies were included that were published up until 17 December 2020.Eligibility was assessed independently by two authors (V.Q.S. and C.F.M.S.; review team members). Meeting reports were considered to be eligible. Disagreement or discrepancies between the review team members were discussed until consensus was reached. When needed, a third author with expertise in the specific research field was consulted.

### 2.3. Data Extraction

Due to the expected heterogeneity of the included articles in terms of disease model, tracer type, and imaging modality, all study characteristics and outcomes were described via a narrative synthesis. No predefined, exact outcome measurements were therefore extracted. Fundamental characteristics such as imaging modality, tumor or cardiovascular disease model (species, cell line), tracer, and tracer class were extracted independently by the review team.

## 3. Results

### 3.1. Study Selection

**Figure 2:**
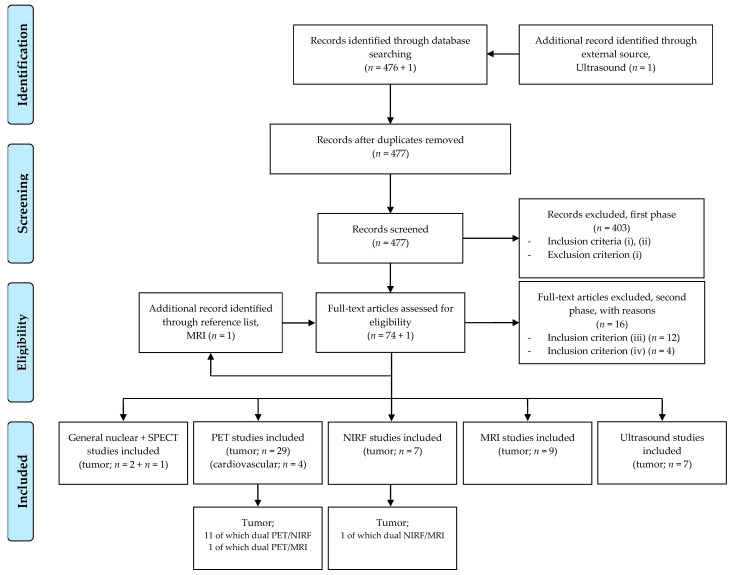
Flow chart demonstrating the study selection process.

The PubMed search on endoglin-based imaging for cancer and cardiovascular diseases revealed a total of 476 articles (Figure 2). Additional studies on MR (*n* = 1) and ultrasound imaging (*n* = 1) in tumor models were found, respectively, via the reference list of an included article and through an external source (reviewer). The selection procedure was split up in two phases. The first selection phase, in which 477 titles and abstracts were screened, focused on inclusion criteria (i) and (ii) and exclusion criterion (i) (Figure 2). After exclusion of 403 articles, 74 + 1 full-text articles were assessed in the second selection phase, focusing on inclusion criteria (iii) and (iv). The resulting 59 articles consisted of 55 studies in tumor models: general nuclear imaging (2), SPECT (1), PET (17), MRI (9), NIRF (6), ultrasound (7), dual PET/NIRF (11), dual PET/MRI (1), and dual NIRF/MRI (1). The remaining four articles used PET to image cardiovascular disease models.

### 3.2. Endoglin-Based Cancer Imaging

#### 3.2.1. Angiogenesis and Tumor(-Associated) Cells

Neovascularization is essential for tumor growth, development, and metastasis [27]. Therefore, the concept of targeting microvessels for diagnostic and therapeutic purposes is rather well established. As compared to other angiogenic targets on endothelial cells, such as vascular endothelial growth factor receptor (VEGFR), endoglin has an up to 10 times higher expression level [28,29,30]. An evident application of endoglin would therefore be noninvasive, in vivo detection of angiogenesis for diagnosis and prediction of tumor progression. This could be effectuated via imaging, by employing an endoglin-targeting tracer (e.g., antibodies, peptides, or nanoparticles), and by the use of labels and corresponding imaging systems. During surgery, real-time imaging with targeting probes and imaging techniques such as NIRF could assist surgeons to identify and completely resect malignant tissue more precisely, while sparing vital surrounding tissues [5]. For both types of imaging applications, important target characteristics include distribution within the tumor and the availability of a specific tracer.

Although the efficacy of endoglin-based whole-tumor imaging could be questioned due to the proclivity of endoglin expression on activated endothelium rather than on malignant cells, similar tracers and novel insights suggest differently. For example, the (pre) clinical results of other neoangiogenesis-based tracers, like cRGD-peptide- (α_v_β_3_ integrin) and vascular endothelial growth factor (VEGF)-targeting antibodies demonstrate efficient whole-tumor imaging [31,32]. Next to its high presence on endothelial cells, expression of endoglin has also been shown on fibroblast-like stromal cells at the invasive fronts of colorectal and prostate cancer [33,34]. Various malignant cell types of epithelial origin show an increase in endoglin expression level, including in primary endometrial cancer, head and neck squamous cell carcinoma (especially in tissue samples from metastatic patients), and metastatic breast cancer cells [35,36,37]. Therefore, those carcinomas would be good candidates for endoglin-based imaging, even when endoglin’s tumor promoting or suppressing role in these settings remains currently undetermined. Based on the endoglin-expressing cells in the tumor microenvironment, i.e., angiogenic endothelial cells, subtypes of fibroblasts, and some malignant epithelial cells, specific endoglin targeting agents have been developed for cancer therapy. The most promising is most likely carotuximab (TRC105) (reviewed in [38]). TRC105 is an endoglin-binding chimeric monoclonal antibody (human/mouse), designed for minimal immunogenicity in patients. Next to therapy, this antibody would be particularly suitable for diagnostic and imaging purposes, owing to its good tolerability, high accumulation, and limited side effects. Moreover, its affinity for human as well as mouse endoglin qualifies TRC105-based tracers for direct preclinical evaluation in mouse models.

Exploitation of endoglin-based imaging of pathological lesions in a (pre) clinical setting, based on compounds developed for therapy, would accelerate a clinically translatable approach. An obvious application would allow for quantification (PET, SPECT) or real-time visualization (NIRF) of neovessels, providing a better alternative to conventional histological MVD determination. Importantly, although current imaging systems may also reveal vascularization, they do not discern between existing vessels and neovasculature. In addition to vascularization, endoglin-based imaging could allow for precise image-guided resections, upon identification of specific tumor types that express endoglin beyond the endothelium or are highly vascularized. For all clinical applications, important criteria include a high lesion‑to-background ratio, minimized toxicity, and an optimized combination of tracer and imaging modality. The latter subject will be explored further below in the context of the current literature on noninvasive, endoglin-based tumor imaging.

#### 3.2.2. Endoglin-Based Nuclear Imaging of Tumors

Nuclear imaging systems, which operate via the detection of radiotracers, have an established role in cancer diagnostics [39]. In short, dedicated radiotracer decay, which results in the emission of positrons or gamma rays, is measured by PET and SPECT scanners respectively, and transformed into an image of the targeted tissue. The most commonly used radiotracer in PET imaging is ^18^F-FDG, a radiolabeled sugar molecule that accumulates in cells with enhanced glucose metabolism, such as cancer cells. For SPECT imaging, a wide variety of tracers are employed that are coupled to radioisotopes, such as ^99m^Tc, ^123^I, and ^111^In. Upon intravenous injection of these radiopharmaceuticals and the use of CT for anatomical referencing, an accurate, contrast-rich, and three-dimensional image of the targeted tissue can be created to (i) reveal and monitor malignant tissues, (ii) portray pathophysiological and molecular processes (e.g., tumor angiogenesis and heterogeneity), and (iii) guide surgical procedures and facilitate image-guided biopsies [40]. Currently, their added value lies primarily within the realm of diagnostics. With regard to surgery, combinations with NIRF imaging have been shown to guide surgical oncologists based on preoperative PET or SPECT maps of various types of tumors (e.g., lymphoma, prostate, breast, and thyroid cancer) [41,42,43,44,45].

Our literature search revealed one endoglin-based SPECT article (Figure 2, Table 1) [46]. The authors assessed the biodistribution, tumor-targeting properties, and in vivo deiodination of both directly and indirectly ^125^I-labeled, anti-CD105 monoclonal antibodies (mAbs) in mice bearing murine melanoma cell line B16F10. Interestingly, they concluded that direct iodination of the anti-CD105 antibodies resulted in swift dehalogenation in vivo, mediated via catabolic processes. In contrast, indirect iodination via a D-KRYRR linker peptide led to reduced dehalogenation, slower clearance, and higher tumor-specific accumulation, demonstrating that the antibody pertained its antigen-specific binding [46].

Despite these interesting results, endoglin-based nuclear imaging research has a widespread preference for PET (Figure 2). Historically, the earliest general endoglin-based nuclear imaging study was reported by Fonsatti and coworkers in 2000, by radiolabeling the anti-endoglin monoclonal antibody MAEND3 with ^125^I and demonstrating specific imaging of breast cancer in a canine model [47]. The earliest and only ex vivo study in human organs was performed in 2004. Adequate imaging was reported by using the anti-CD105 monoclonal antibody E9 in freshly excised kidneys from seven patients with renal cell carcinoma [48]. The ^99m^Tc-labeled mAb E9 was perfused through the kidney and subsequently detected via immunoscintigraphs, demonstrating to match the pre- and post-surgically determined tumor locations by MRI and histopathology [48]. Seven years later, Cai and coworkers translated this concept to PET with the TRC105 antibody, by specifically studying the in vivo properties of two commonly employed chelators: 1,4,7,10-tetraazacyclododecane-1,4,7,10-tetraacetic acid (DOTA) and 1,4,7-triazacyclononane-1,4,7-triacetic acid (NOTA) [49]. In a 4T1 murine breast cancer mouse model, they demonstrated that the choice of chelator for conjugation of TRC105 with ^64^Cu did not affect the antibody’s specificity or binding capacities. PET imaging demonstrated that ^64^Cu-NOTA-TRC105 displayed increased stability compared to ^64^Cu-DOTA-TRC105, illustrated by decreased liver uptake with equal targeting competence [49]. Additional studies in the 4T1 in vivo model showed that the Fab and F(ab’)_2_ fragments of TRC105 coupled to ^64^Cu were taken up faster but at a lower peak (t = 24 h, t = 48 h, respectively) compared to the intact antibody ^64^Cu-NOTA-TRC105 [50,51]. In 2012, the same group demonstrated that ^66^Ga-NOTA-TRC105 had similar binding affinity and specificity compared to unlabeled TRC105 [52]. Moreover, this PET-tracer was able to image breast cancer in the 4T1 murine model [52]. A follow-up study in the same model showed that TRC105 could be labeled with ^89^Zr, by employing the chelator desferrioxamine B, to effectively visualize the 4T1 tumor in vivo [53]. Another chelator, diethylenetriaminepentaacetic acid (DTPA), was used in the aforementioned breast cancer mouse model to conjugate TRC105 with various yttrium isotopes (^86/90^Y) [54]. ^86^Y is a relatively new isotope for PET imaging, and the validation of ^86^Y-DTPA-TRC105 as a functional in vivo PET imaging tracer is a great example of the ever-expanding field of promising radioisotopes [54].

Next to breast cancer models, mice bearing human glioblastoma U87MG (EGFR/CD105^+/+^) tumors were used to examine the potential of endoglin-based imaging. A bispecific immunoconjugate, consisting of two Fab fragments against EGFR and CD105, was used to enhance specificity to desired targets and subsequently improve imaging [55]. In this study, dual targeting increased both tumor-specificity and -affinity of the immunoconjugate, resulting in separate ^64^Cu‑mediated PET (Table 1: PET) and ZW800-mediated NIRF imaging of glioblastomas in vivo (Table 1: NIRF) [55]. Comparably, dual targeting of human pancreatic adenocarcinomas (BXPC-3 cell line) in an orthotopic mouse model was achieved with a ^64^Cu-NOTA-conjugated heterodimer of anti-tissue factor and anti-endoglin Fab [56].

The earliest studies on endoglin-based imaging with the use of nanomaterials described the application of ^64^Cu and ^66^Ga radiolabeled, ultra-high surface area TRC-105-nanographene as potent in vivo imaging tracer for the 4T1 breast tumor vasculature [57,58,59]. The authors reported excellent specificity, minimal extravasation, and appropriate tracer stability. Notably, toxicity and safety issues with the graphene family of nanomaterials have been studied with contrasting results, ranging from chronic organ injury to inconsequential long-term retainment upon polyethylene glycol (PEG) functionalization [60,61]. Via a similar concept, the regularly applied ^64^Cu-TRC105 combination conjugated with the nanomaterial reduced graphene oxide (RGO) has been found to target tumor neovasculature in the 4T1 mouse model [62]. RGO is an effective photothermal agent which can be employed for tumor ablation as well as imaging purposes [63]. An alternative approach is the use of targeted gold nanoparticles (AuNPs) for PET imaging of melanoma xenografts in C57BL/6J mice (B16F10 cell line) [64]. ^89^Zr-labeled anti-CD105 monoclonal antibodies were conjugated to plasma-polymerized allylamine (PPAA)-coated AuNPs, intravenously injected in the mice, and subsequently assessed for biodistribution via PET imaging. Compellingly, no imaging-limiting effects were found upon AuNP-antibody conjugation, whereas high tumor-to-tissue ratio and tumor selectivity allowed for effective cancer detection [64]. The application of AuNPs in the field of imaging is promising, especially due to their modifiability, biodistribution, and possibility of photothermal therapy [64,65].

Radiolabeled unimolecular micelles represent another nanoplatform for endoglin-based PET imaging. The outer layer of these micelles is a hydrophilic shell, often consisting of PEG, allowing for the transport of hydrophobic drugs. Unimolecularity ensures stability in the circulation, in contrast to multimolecular micelles which disassemble as a consequence of dilution [66]. Two different types of unimolecular micelles that were equipped with TRC105, NOTA, and ^64^Cu were reported: (i) unimolecular micelles formed by dendritic amphiphilic block copolymers poly(amidoamine)–poly(l-lactide)-b-poly(ethylene glycol) (PAMAM-PLA-PEG) and (ii) brush-shaped amphiphilic block copolymer poly(2-hydroxyethyl methacrylate) (PHEMA) with side chains consisting of poly(l-lactide)-PEG (PLLA-PEG) [66,67]. The first type of unimolecular micelle could release a cargo of the cytotoxic agent doxorubicin upon sensing tumor-specific pH changes in the 4T1 breast cancer model in vivo [66]. Combined with satisfactory PET capacities, these micelles could be employed in theragnostic approaches. The second type of unimolecular micelles also showed pH-controlled, tumor-specific doxorubicin release, competent PET-mediated tumor visualization, and equal tumor distribution in the same in vivo model [67].

The tumor-specific characteristics of the anti-endoglin antibody TRC105 have also been combined with biocompatible, radiolabeled PEGylated mesoporous silica (mSiO_2_) nanoparticles (^64^Cu-NOTA-mSiO_2_-PEG-TRC105), which aggregated specifically at 4T1 breast tumor sites in mice after intravenous administration [68]. This accumulation was reported to be mediated via both (i) the enhanced permeability and retention effect (EPR), i.e., accumulation of particles at tumor sites due to large-pored neovasculature, and (ii) specific binding to CD105 expressing vasculature. In general, while interesting features of mesoporous silica nanoparticles (MSNs), such as their easily modifiable porous structure and large surface area, allow for interesting theragnostic applications, concerns regarding unwanted nonspecific reticuloendothelial-system-mediated uptake and reduced elimination have limited their widespread application [69]. In a second iteration, biodegradable MSNs (bMSNs), capable of carrying multiple loads and of self-destruction upon cargo release, were published [70]. In the 4T1 breast cancer model, PEGylated, chelator-free, ^89^Zr-TRC105-labeled bMSNs allowed for stable and tumor-specific PET imaging [70]. In 2018, this research group also achieved to combine hollow MSNs (hMSNs) with quantum dots (QD; fluorescent emitter) into a so-called yolk-shell hybrid nanosystem for effective dual PET and NIRF imaging of 4T1 tumors [71]. Moreover, they showed the feasibility of tumor vasculature imaging with an upconversion nanoparticle (UCNP)@^89^Zr-hMSN-PEG-TRC105 tracer at 980 nm [72]. The same study also demonstrated that general synthesis of similar ^89^Zr‑labeled hMSNs, with internal cores of inorganic functional nanoparticles and superparamagnetic iron oxide nanoparticles (SPIONs) was feasible. SPION cores can be used in the field of MRI and will be elaborately discussed in the next section. In the context of dual PET and MR imaging, PEGylated manganese oxide (Mn_3_O_4_) nanoparticles were developed and conjugated with TRC105 and ^64^Cu, which exhibited low toxicity and high specificity for CD105 positive vasculature in 4T1 breast tumors [73].

#### 3.2.3. Endoglin-Based MR Imaging of Tumors

MRI is a powerful imaging modality that makes use of strong magnetic fields and radiofrequency waves to capture images of the human body with high spatial and temporal resolution. MRI contrast agents traditionally differ in their indirect and nonlinear behavior from the direct and linear principles of contrast agents used for CT or the gamma ray-emitting isotopes for nuclear scintigraphy. These differences are based on the distinct magnetic properties and interactions of ^1^H protons toward the applied MRI contrast agent; as such, the dose of the contrast agent and used MRI sequence determines whether either T1 shortening (positive) or T2 shortening (negative) contrast effects are predominant. Typical positive MRI contrast agents are Gd- or Mn-containing paramagnetic chelates, while Fe-containing superparamagnetic crystals of nanoparticles are referred to as negative or susceptibility contrast agents. The recent increasing demand for sensitive MRI contrast agents has initiated the development of hybrid T1/T2 dual agents that combine the imaging properties of both T1 and T2 in a synergistic manner [74]. T1 and/or T2 properties of endoglin-based MR tracers are indicated in Table 1. SPIONs are commonly used in (preclinical) endoglin-based MR imaging, as they can be readily modified to bind tumor-specific antibodies or to carry drugs, serving as a theragnostic platform [75], (reviewed in [76]).

Dassler et al. optimized SPIONs with a biocompatible polyacrylic acid (PAA) coating conjugated with a monoclonal antibody against mouse endoglin [77]. Initially, they demonstrated via autoradiography that ^59^Fe-radiolabeled αCD105-PAA-SPIONs could accumulate at endoglin-expressing, highly vascularized teratocarcinomas in mice (F9 teratoma model). However, upon MR imaging of αCD105-PAA-SPIONs in the same model, limitations in signal detection sensitivity hampered tumor-specific visualization. The authors argued that improving the imaging properties of SPIONs would be insufficient in this case, considering the limitations of main MRI sequences and field strength [77].

Using a different strategy, the detection of early tumor angiogenesis was enabled in rats bearing extracerebral gliomas (F98 cell line) [78]. By entrapping the MRI contrast agent gadolinium-diethylenetriamine pentaacetic acid (Gd-DTPA) into PEG-coated liposomes, the researchers created a versatile foundation for a targeting vehicle. After conjugation with an anti-CD105 monoclonal antibody, the anti-CD105-Gd-liposomes were injected intravenously into the animals. The signal intensity of the liposomes primarily granted visualization of the tumor-specific vasculature but was also sufficient to enable accurate whole-tumor imaging for up to 120 min [78]. In a similar approach, tumor margins in a glioma rat model (C6 cell line) were effectively delineated by targeting endoglin with paramagnetic liposomes conjugated to monoclonal anti-CD105 antibodies [79]. These sterically stabilized, paramagnetic liposomes were intravenously injected into the animals and showed effective tumor imaging capabilities.

The applicability of single-walled carbon nanotubes (SWCNTs) as a theragnostic, endoglin-targeting MRI tracer was demonstrated by tagging polyvinylpyrrolidone (PVP)-functionalized SWCNTs with SPIONs and conjugating them with an anti-mouse endoglin antibody [80]. After loading the nanotubes with doxorubicin, the magnetic nanoparticles were administered intravenously to mice bearing luciferase-expressing 4T1 breast cancer cells. The researchers had previously shown that adequate efficiency of tumor-specific accumulation of the modified SWCNTs was feasible via MRI in the murine 4T1 breast cancer model with good biocompatibility [81]. Interestingly, an external flexible high-energy magnet was used to guide the delivery of the tracers to the known tumor site [80,81]. Comparable MR imaging results with identical SWCNTs and external magnets were obtained in a subsequent study focusing on lung metastases in the 4T1 model [82].

Nanomicelles have also been adopted for endoglin-based tumor MR imaging. Shelled nanoprobes consisting of the CD105-specific peptide CL 1555 (amino acid sequence: AHKHVHHVPVRL), a poly(ε-caprolactone)-block-poly(ethylene glycol) amphiphilic copolymer, and a core comprising manganese ferrite (MnFe_2_O_4_) nanoparticles, were able to induce high contrast MR imaging of tumor vascular endothelial cells in vitro [83]. In another study, the same authors designed an anti-CD105-conjugated nanoparticle hybrid based on a SPION core (maghemite, γ-Fe_2_O_3_) and a golden shell (Au) with the purpose of MR imaging of tumor angiogenesis [84]. Gold-coating reduces toxicity of the iron oxide core and decreases aggregation, adding to the stability of the hybrid on top of the PEGylation process [85,86]. MRI signal was detected 60 min after intravenous injection of the nanomicelles in BALB/c mice bearing the human breast adenocarcinoma MDA-MB-231 cell line. A positive correlation was found between the relative signal intensity and histologically assessed MVD based on CD105 [84].

Similarly, magnetic endoglin-specific aptamer nanoprobes based on modified magnetic carboxymethyl chitosan (CMCS) nanoparticles (aptamer-Fe_3_O_4_@CMCS) were generated to target subcutaneous H22 hepatocellular carcinomas in mice [87]. The results showed that tumor neovascularization could be accurately imaged with low toxicity and adequate biocompatibility by using aptamer-Fe_3_O_4_@CMCS for MRI.

#### 3.2.4. Endoglin-Based Near-Infrared Fluorescence Imaging of Tumors

Like for targeted PET, SPECT, and MRI, most targeted tracers for NIRF-based imaging consist of dyes conjugated to peptides, (derivatives of) monoclonal antibodies, aptamers consisting of single chain DNA/RNA, or of other carriers equipped with these tracing elements, such as nanoparticles, viruses, or cell-derived vesicles [88]. NIRF imaging further depends on a fluorescent dye and a near-infrared light source with a specific wavelength to illuminate the tissue of interest. Electrons in the atoms of the fluorescent dye react on this excitation light by transferring from the ground state to the excited state. Upon return to the ground state, these electrons emit a near-infrared signal with a longer wavelength, which can be visualized with a camera system. Optical imaging with NIRF light operates beyond the spectrum of visible light (700–900 nm), which demands the use of dedicated near-infrared camera systems and filter settings. The limited tissue penetration, real-time view, and absence of radiation make NIR light-based imaging ideal for use during surgery. Moreover, the imaging tracers and camera systems are relatively cheap and manageable [5].

**Figure 3:**
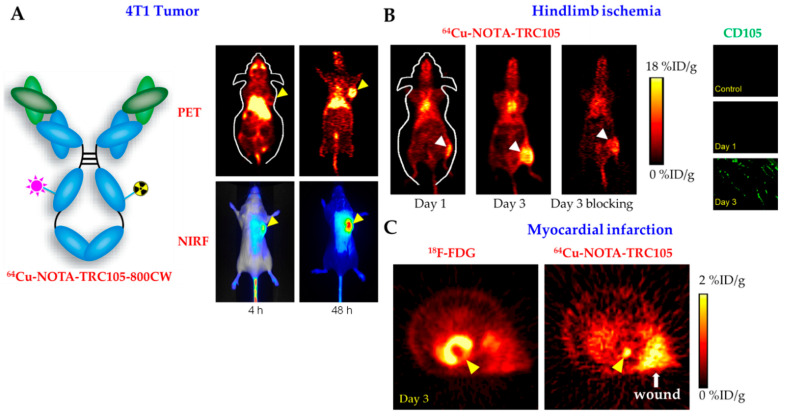
Endoglin-based imaging in three different animal models. (**A**) Dual-modality positron emission tomography (PET) and near-infrared fluorescence (NIRF) imaging of CD105 in the 4T1 murine breast cancer model with ^64^Cu-NOTA-TRC105-800CW. Arrowheads indicate the 4T1 tumor. Adapted from Zhang et al., 2012 [104]. (**B**) ^64^Cu-NOTA-TRC105 PET imaging of CD105 in the murine hindlimb ischemia model. Tracer uptake peaked in the ischemic hindlimb (arrowheads) at around 3 days after surgery. CD105-specificity of tracer uptake was confirmed by successful blocking experiments and strong CD105 staining. Adapted from Orbay et al., 2013 [150]. (**C**) In a rat model of myocardial infarction, confirmed by the gap in the 18F-FDG PET image (arrowhead), ^64^Cu-NOTA-TRC105 PET allowed for noninvasive imaging of CD105 in vivo (arrowhead). The wound was also delineated by the tracer since it has a high level of angiogenesis and CD105 expression. Adapted from Orbay et al. 2013 [152].

Although the use of camera systems may seem cumbersome and distracting in the operating theatre, it has rapidly become common practice, especially during minimally invasive interventions such as laparoscopy, sentinel lymph node procedures, and robot assisted surgery. A tumor-representing NIRF signal is superimposed on the signal captured by a visible light camera, providing contrast between healthy and malignant tissue on a monitor. As shown in Figure 3, small lesions can be clearly recognized. In various clinical studies, tumor-specific NIRF dye-labeled antibodies have been used in oncologic intraoperative settings (e.g., colorectal liver metastases, pancreatic cancer, and ovarian cancer) [89,90,91].

An important characteristic of NIRF is the inability of humans to perceive its emission and excitation wavelengths, ensuring its on-demand availability upon activation of the accessory camera without interfering with regular, visible light-based surgical workflows. Moreover, NIRF imaging has low tissue-autofluorescence and is barely affected by tissue scattering and absorption, establishing its favorable tumor-to-background ratio and accurate target delineation. The real-time imaging capabilities of NIRF in combination with its high spatial and temporal resolution make it an attractive imaging modality for IGS. The classical near-infrared wavelength window (NIR-I) allows for imaging depths of up to 1 cm deep, depending on tissue composition. Despite this limited penetration depth, it has already been clinically applied. More recently, a second NIR wavelength window (NIR-II, 1000–1700 nm) has gained attention due to its improved contrast, resolution, and imaging depth capabilities (1–3 cm) [92,93]. An interesting development is the implementation of nanotechnologies in this field, with the creation of NIR-I-to-NIR-II nanomaterials that absorb light in the NIR-I window and emit sharp fluorescent signals in the NIR-II window (e.g., single-walled carbon nanotubes, quantum dots, polymer nanoparticles) (reviewed in [94]).

A challenge in the field of fluorescence-guided surgery is the transition from preclinical and early phase clinical trials into phase 2–3 studies, to demonstrate clinical benefit [95]. Next to procedural challenges such as the need of motivated and instructed clinicians, initiating competent trial teams, and setting up adequate protocols, overcoming technical problems such as appropriate matching of camera system and imaging agent is crucial. Moreover, it should be kept in mind that, although NIRF laparoscopic surgery has some distinct advantages compared to NIRF open surgery in terms of reduced background light and imaging distance, its imaging capacity is lower due to safety issues related to excitation laser power [95].

Early experiments with endoglin-based NIRF imaging started in 2009, when an in vitro model based on human umbilical vein endothelial cells (HUVECs) was employed to simulate angiogenic and physiological vasculature and demonstrate effectiveness of molecular imaging of angiogenesis [96]. Although the authors were able to image angiogenesis by using a planar NIRF imager and endoglin- and VEGFR2-specific contrast agents, an important limitation was the lack of additional cell types serving as a tumor environment. Nonetheless, an interesting observation was that the highest NIRF signal was that of endoglin in proliferating endothelial cells, while VEGFR2 fluorescent signal could not be observed [96]. Two years later, the anti-endoglin chimeric antibody TRC105 was conjugated to the NIRF dye IRDye 800CW, and subsequently injected intravenously in 4T1 breast cancer-bearing mice [97]. The specificity of the tracer was confirmed using blocking experiments, control antibodies, and histology. Moreover, the authors claimed an 85% 800CW-TRC105 conjugation yield without any alterations to the binding affinity to endoglin [97].

In 2018, using M13 phage display, 13 peptides directed against endoglin were identified [98]. Peptides are associated with improved heat stability and tumor penetrative capabilities compared to antibodies and are cheaper to develop and produce [99]. Peptide nABP296 was identified as having the highest affinity for the endoglin-expressing MNNG/HOS cell line (human osteosarcoma) and was conjugated with the fluorescent dye fluorescein-5-isothiocyanate (FITC). One hour after intravenous administration of FITC-nABP296 in subcutaneous MNNG/HOS xenografted mice, specific fluorescent signal in the tumor was shown. Sections of an osteosarcoma patient, that were stained using nABP296 and an anti-CD105-antibody, demonstrated overlapping specificity of both proteins for osteosarcomas ex vivo [98].

In the field of endoglin-based NIRF imaging, various groups have been focusing on improving specificity via the use of nanomaterials, like activatable liposomes [100]. Liposomes are nanocarriers that are especially under investigation in the context of drug delivery because they are easily modifiable, biocompatible, and have high payload capacity (reviewed in [101]). Upon encapsulating the liposomes with the NIRF dye DY-676-COOH and conjugation with antibody fragments against endoglin (END-IL), it was shown that NIRF imaging of xenografted human MDA-MB-231 breast cancer cells and HT1080 fibrosarcoma cells was feasible in mice [100]. NIRF imaging capacity was dependent on uptake and degradation of the liposomes by the desired target cells, based on self-quenching of DY-676-COOH at high concentrations. Interestingly, whereas the human (h)END-IL principally accumulated at the tumor site, the mouse (m)END-IL tended to accumulate in the liver, kidneys, lungs, and vasculature of the mice. Validation via confocal microscopy on harvested tissues showed that hEnd-IL predominantly bound to the tumor cells, while mEnd-IL was more specific for murine vasculature. In addition to tumor cells, the authors also indicated that the probes could be taken up by phagocytes, thereby possibly targeting phagocytic cells like tumor associated macrophages [100]. The liposomes were recently adjusted to simultaneously target fibroblast activation protein (FAP) and endoglin via the addition of specific single chain antibody fragments (Bi-FAP/mEND-IL) [102]. Similar to the previous study, mice bearing a xenografted fibrosarcoma (HT1080-hFAP, high FAP expression, large vasculature) or breast tumor (MDA-MB-231, no FAP expression, neovasculature) were injected with the bispecific liposomes and subsequently imaged [102]. The dye cargos were adequately delivered at, respectively, the FAP-expressing cells and neoangiogenic endothelium [102]. Similar results have been obtained by adding the antibody anti‑CD105 ILp to the surface of liposomes, demonstrating effective imaging of tumor vasculature in human MDA-MB-231 breast cancer-bearing mice [103].

This paragraph will discuss studies that combined NIRF with PET and/or MR imaging. In 2012, Cai and his group demonstrated that TRC105 labeled with both a NIRF dye (800CW) and ^64^Cu could be employed to successfully image 4T1 breast tumors in vivo, showing good overlap between the two imaging modalities (Figure 3A) [104]. In vitro experiments demonstrated that the dual-labeled antibody bound to HUVECs with high specificity, yet not to 4T1 cells which lack CD105 expression. In the same year, comparable results were found with a combination of TRC105, 800CW, and a different PET-isotope, ^89^Zr, in the aforementioned in vivo 4T1 breast cancer mouse model [105]. A meeting report and two articles showed that both the ^64^Cu- and ^89^Zr, dual-labeled (800CW) antibodies were capable of primarily imaging angiogenesis and small tumor nodules in the lung metastatic 4T1 breast cancer model, supported by various in vitro and ex vivo experiments [106,107,108]. Importantly, NIRF and not PET imaging struggled to properly delineate the lung metastases, which was attributed to the limited tissue penetration of NIRF and the relatively deep position of the tissue [107]. By using a heterodimer of CD105 and tissue factor Fab antibody fragments, labeled simultaneously with ZW800 and ^64^Cu or single-labeled with ^64^Cu, the same group demonstrated the feasibility of in vivo NIRF and PET imaging of human BxPC-3 and PANC-1 pancreatic tumors [56,109]. In the domain of multimodality-imaging, various effective endoglin-targeting nanoparticles in the context of the 4T1 breast cancer mouse model have been reported, including (i) small copper sulfide nanoparticles (NIRF absorption and extinction properties), surrounded by mesoporous silica nanoshells and labeled with TRC105 and ^64^Cu, (ii) hMSNs, loaded with doxorubicin and ZW800 and conjugated with TRC105 and ^64^Cu, and (iii) red fluorescent zinc oxide nanoparticles conjugated with ^64^Cu and TRC105 [110,111,112]. In general, the flexibility of these nanoparticles allows for the combination of multiple imaging techniques such as MRI and NIRF. In this context, SMMC-7721 hepatic cellular carcinoma xenografts were effectively imaged with both imaging modalities by employing an endoglin‑targeted aptamer–dendrimer combination that was conjugated with a paramagnetic agent (Gd-DTPA) and a near-infrared fluorophore (IR783) [113].

#### 3.2.5. Endoglin-Based Ultrasound Imaging of Tumors

Imaging based on ultrasound functions via the transmission of non-ionizing, high-frequency sound waves through target tissue. These soundwaves are reflected differently by various tissue types based on their density (acoustical impedance) and are processed into greyscale, real-time images with good contrast. Developments over several decades have culminated in various improved techniques as compared to the original two-dimensional ultrasound images, including three- and four-dimensional acquisition, blood flow measurements via color doppler, and contrast-enhanced ultrasound (CEUS) imaging via the use of microbubbles. Next to the obvious advantage of its non-harmful nature, ultrasound is relatively cheap, readily available and fast, has a manageable and portable size compared to MR and nuclear imaging modalities, and allows for simultaneous visualization of morphology and pathophysiological processes. However, when considering utilizing ultrasound in any context, one must keep in mind that effective image acquisition demands training and experience. Moreover, the emitted sound waves are inept to travel effectively through echodense structures (e.g., bone, thick vascular calcifications), resulting in acoustic shadows that impair the viewing field behind these solid structures. Nonetheless, ultrasound imaging is a promising modality in cardiovascular and tumor imaging, as indicated by its clinical adoption in diseases such as aortic aneurysms, atherosclerotic obstructions, and ovarian cancer [114,115,116].

Microbubbles are shelled gas bubbles with a diameter of 0.5–10 μm, which exceptionally react to ultrasonic waves by expansion and contraction. This response leads to emission of detectable signals, allowing for distinct imaging by the ultrasound transducer and increasing (targeted) contrast. Moreover, a so-called destruction pulse can be used to increase targeted contrast or induce content-release (e.g., therapeutic agents) and modifications to the structure of microbubbles allow for increased theragnostic specificity and local drug release (reviewed in [117]). It is important to keep in mind that the diameter of microbubbles only allow for targeting of intravascular markers, although nanobubbles have been proposed as extravascular targeting agents with the capability of traversing (leaky) vascular endothelium because of their size (<200 nm) [118]. Notably, all microbubble constructs reported in the paragraphs below were functionalized with (strept)avidin, a protein foreign to humans with potential immunogenic risks, allowing for proof-of-principle studies but impairing clinical use.

The first use of targeting microbubbles to endoglin was reported in an in vitro culture of endothelial cells (bEND.3 cell line) [119]. Avidin–biotin interaction was employed for noncovalent coupling of rat anti-mouse monoclonal anti-endoglin antibodies (MJ7/18) to microbubbles. Avidin was incorporated into the shell of perfluorocarbon-exposed dextrose albumin (PESDA) microbubbles via sonication. FITC-conjugated biotinylated monoclonal antibodies were linked to the microbubbles (anti-CD105-avidin-PESDA-Microbubble) and subsequently incubated with cultured endothelial cells and 3T3 fibroblasts, demonstrating specific binding to the former but not to the latter subtype [119]. In a subsequent study, an in vivo subcutaneous mouse model of pancreatic cancer (Pan02 cell line) was used [120]. After 5 weeks of treatment with either the chemotherapeutic drug gemcitabine or a control agent, video-intensity of anti-CD105-avidin-PESDA-Microbubbles was significantly lower in the gemcitabine group, as supported by comparable results obtained with immunofluorescence-assessed endothelial cell marker expression (CD105 and VEGFR2) and MVD determination. In short, video-intensity is calculated by subtraction of background video-intensity from the mean video intensity generated by targeted microbubbles [121,122]. In the same article, the authors used microbubbles conjugated with anti-VEGFR2. They reported that no toxicity was observed during or after all ultrasonic applications [120]. In another study, streptavidin-biotin binding chemistry to fabricate microbubbles targeted to biotinylated rabbit anti-mouse α_v_β_3_ integrin, mouse VEGFR2, mouse endoglin, and a control IgG was reported [123]. The microbubbles contained streptavidin moieties in their lipid shell, enclosing perfluorocarbon. Ovarian adenocarcinoma (SKOV3)-, breast adenocarcinoma (MDA-MB-361)-, and pancreatic adenocarcinoma (MiaPaCa2)-bearing mice were injected with four boluses (interval of 30 min) that contained all four microbubble types. Real-time, longitudinal CEUS of all three targeted microbubbles showed effective quantification of angiogenic marker expression via video-intensity in all three cancer models. Especially for endoglin, good correlation was found between ex vivo immunoblotting analyses and targeted CEUS imaging signal (ρ = 0.88) [123]. In a similar study, the video-intensity of lipid-shelled microbubbles coupled via streptavidin-biotin binding chemistry to anti-mouse rat integrin α_v_, VEGFR2, endoglin, and a control IgG was evaluated in a B16-F10 melanoma mouse model [124]. The mice received every day either sorafenib, a multikinase inhibitor which inhibits angiogenesis [125,126], or a control solution, and underwent CEUS imaging at days 0 and 3. Calculated differential targeted enhancement (dTE), analogous to the previously described video-intensity, revealed that endoglin-conjugated microbubbles displayed significantly increased signal intensity as compared to anti-integrin α_v_ and anti-VEGFR2 microbubbles. Moreover, endoglin microbubbles demonstrated increased signal intensity in the control treated group as compared to the mice that received sorafenib [124]. CD105-targeted perfluorocarbon-containing lipid-shelled microbubbles using avidin-biotin binding chemistry (anti-CD105-avidin-Microbubble) were employed in the context of subcutaneous U87MG glioblastoma-bearing mice [127]. After in vitro immunofluorescence confirmed specificity of the CD105-targeted microbubbles to CD105 positive endothelial cells (MS1 cell line) compared to 4T1 breast cancer cells, adequate positive correlation in the glioblastoma model between ex vivo CD105 immunofluorescence and in vivo ultrasound dTE (ρ = 0.86) was shown. In a model of HepG2 hepatoblastoma-bearing nude mice, microbubbles conjugated with anti-mouse anti-endoglin antibodies had a significantly higher dTE than microbubbles conjugated with an isotype IgG in the vasculature of the xenografted tumor [128]. Comparably, CD105-targeted microbubbles were studied in the context of two separate xenografted cholangiocarcinoma mouse models (TFK-1, EGI-1 cell lines) and demonstrated significant capacity to bind to both tumors compared to isotype control microbubbles [129].

Intriguing research that is beyond the scope of this review is the work by the group of Foster, proving that endoglin-targeting microbubbles can be used to image the vasculature of living mouse embryos [130,131].

**Table 1:** Overview of current endoglin-based tumor imaging studies, categorized by imaging modality, disease model, and imaging agent.

Imaging Principle	Specific	Model (h) = Human, (m) = Murine	Imaging Agent	Class
Nuclear imaging (*n* = 20)	General (*n* = 2)	Dogs with spontaneous mammary tumors	^125^I-MAEND3 [47] ^a^	a. Antibody
		Human renal cell carcinoma patients * (excised human kidneys)	^99m^Tc-E9 mAb [48] ^a^	
	SPECT (*n* = 1)	B16F10 (m) melanoma model	^125^I-anti-CD105 mAb [46] ^a^	a. Antibody
	PET (*n* = 17)	4T1 (m) breast cancer mouse model	^64^Cu-NOTA-TRC105 and ^64^Cu-DOTA-TRC105 [49] ^a^ ^64^Cu-TRC105-Fab [50] ^b^ ^64^Cu-TRC105-F(ab’)_2_ [51] ^b^ ^66^Ga-NOTA-TRC105 [52] ^a^ ^89^Zr-Df-TRC105 [53] ^a^ ^86^Y-DTPA-TRC105 [54] ^a^ ^64^Cu-NOTA-nanographene-TRC105 [57,58] ^c^ ^66^Ga-NOTA-nanographene-TRC105 [57,59] ^c^ ^64^Cu-NOTA-RGO-TRC105 [62] ^c^ ^64^Cu-NOTA-PAMAM-PLA-PEG-TRC105 [66] ^c^ ^64^Cu-NOTA-PHEMA-PLLA-PEG-TRC105 [67] ^c^ ^64^Cu-NOTA-mSiO_2_-PEG-TRC105 [68] ^c^ ^89^Zr-bMSN-PEG-TRC105 [70] ^c^	a. Antibody b. Fab fragment c. Nanoparticle
		U87MG (h) glioblastoma (EGFR/CD105^+/+^) mouse model	^64^Cu-NOTA-(anti-CD105 and anti-EGFR Fab) [55] ^b^	
		BxPC-3 (h) pancreatic tumor mouse model	^64^Cu-NOTA-(anti-CD105 and anti-TF Fab) [56] ^b^	
		B16F10 (m) melanoma mouse model	^89^Zr-anti-CD105-AuNP-PPAA [64] ^c^	
MRI (*n* = 9)		4T1 (m) breast cancer mouse model	Anti-CD105-PVP-SWCNT-SPION [80,81] ^c^, T2	c. Nanoparticle d. Peptide
		4T1 (m) lung metastases mouse model (breast cancer)	Anti-CD105-PVP-SWCNT-SPION [82] ^c^, T2	
		MDA-MB-231 (h) breast cancer mouse model	Anti-CD105-PEG- (Fe_2_O_3_/au nanoparticle) [84] ^c^, T2	
		F9 (m) teratoma mouse model	αCD105-PAA-SPION [77] ^c^, T2	
		F98 (m) glioma rat model	Anti-CD105-Gd-(PEGylated liposomes) [78] ^c^, T1	
		C6 (m) glioma rat model	Anti-CD105-Gd-(paramagnetic liposomes) [79] ^c^, T1	
		Tumor vascular endothelial cells (coculture, HUVEC: MDA-MB-231 (h); 1:5) (in vitro)	CL 1555-PEG-MnFe_2_O_4_ [83] ^c,d^, dual T1/T2	
		H22 (m) hepatocellular carcinoma mouse model	Aptamer-Fe_3_O_4_@CMCS [87] ^c^, T2	
NIRF (*n* = 6)		4T1 (m) breast cancer mouse model	800CW-TRC105 [97] ^a^	a. Antibody b. Fab fragment c. Nanoparticle d. Peptide
		MNNG/HOS (h) osteosarcoma mouse model	FITC-nABP296 [98] ^d,^**	
		MDA-MB-231 (h) breast cancer and HT1080 (h) fibrosarcoma mouse models	End-IL-Liposomes-DY-676-COOH [100] ^c^ Bi-FAP/mEND-IL liposomes-DY-676-COOH [102] ^c^	
		MDA-MB-231 (h) breast cancer mouse model	Anti-CD105 ILp-liposomes [103] ^c^	
		U87MG (h) glioblastoma (EGFR/CD105^+/+^) tumors	ZW800-NOTA-(anti-CD105 and anti-EGFR Fab) [55] ^b^	
Ultrasound (*n* = 7)		bEND.3 endothelial cells (in vitro)	Anti-CD105-avidin-PESDA-Microbubble [119] ^e^	e. Microbubble
		Pan02 (m) pancreatic cancer mouse model	Anti-CD105-avidin-PESDA-Microbubble [120] ^e^	
		SKOV3 (h) ovarian adenocarcinoma mouse model	Anti-CD105-streptavidin-Microbubble [123] ^e^	
		MDA-MB-361 (h) breast adenocarcinoma mouse model	Anti-CD105-streptavidin-Microbubble [123] ^e^	
		MiaPaCa2 (h) pancreatic adenocarcinoma mouse model	Anti-CD105-streptavidin-Microbubble [123] ^e^	
		B16-F10 (m) melanoma mouse model	Anti-CD105-streptavidin-Microbubble [124] ^e^	
		U87MG (h) glioblastoma mouse model	Anti-CD105-avidin-Microbubble [127] ^e^	
		HepG2 (h) hepatoblastoma mouse model	Anti-CD105-streptavidin-Microbubble [128] ^e^	
		TFK-1 (h) and EGI-1 (h) cholangiocarcinoma mouse model	Anti-CD105-streptavidin-Microbubble [129] ^e^	
Dual imaging (*n* = 13)	PET/NIRF (*n* = 11)	4T1 (m) breast cancer mouse model	^64^Cu-NOTA-TRC105-800CW [104] ^a^ ^89^Zr-Df-TRC105-800CW [105] ^a^ ^64^Cu-CuS@MSN-TRC105 [110] ^c^ ^64^Cu-hMSN-TRC105-ZW800 [111] ^c^ ^64^Cu-NOTA-QD@hMSN-PEG-TRC105 [71] ^c,^** UCNP@^89^Zr-hMSN-PEG-TRC105 [72] ^c^ ^64^Cu-NOTA-ZnO-TRC105 [115] ^c^	a. Antibody b. Fab fragment c. Nanoparticle
		4T1 (m) lung metastatic mouse model (breast cancer)	^89^Zr-Df-TRC105-800CW [106,107] ^a^ ^64^Cu-NOTA-TRC105-800CW [108] ^a^	
		BxPC-3 (h) and PANC-1 (h) pancreatic tumor mouse models	^64^CU-NOTA-(anti-CD105 and anti-TF Fab’ immunoconjugate)-ZW800 [112] ^b^	
	PET/MRI (*n* = 1)	4T1 (m) breast cancer mouse model	^64^Cu-NOTA- Mn_3_O_4_@PEG [73] ^c^	c. Nanoparticle
	NIRF/MRI (*n* = 1)	SMMC-7721 (h) hepatic cellular carcinoma mouse model	Gd-DTPA-aptamer-dendrimer-IR783 [113] ^c^	c. Nanoparticle

* Only ex vivo study in human organs included, ** the absorption/emission spectra of FITC (494/518 nm) and some quantum dots officially fall outside the range of NIRF.

### 3.3. Endoglin-Based Imaging of Cardiovascular Diseases

#### 3.3.1. Imaging of Angiogenesis in Cardiovascular Diseases

Besides the crucial role for angiogenesis in tumor progression, angiogenic processes are involved in multiple cardiovascular diseases, including atherosclerosis (peripheral and coronary artery disease, vein graft disease) and aortic aneurysms (both thoracic and abdominal) [132,133,134,135,136,137]. Endoglin/CD105 has a high sensitivity for the detection of microvessels in atherosclerotic plaques, is involved in the pathophysiology of atherosclerotic lesions, is upregulated in aortic aneurysms, and contributes to shear-induced collateral artery growth [138,139,140]. The combination of the regulatory role of endoglin in intraplaque angiogenesis together with the fact that intraplaque angiogenesis is increasingly related to plaque instability, supports the quantification of plaque destabilization by means of endoglin-based imaging [134,141,142,143]. With such imaging modalities, the identification of unstable plaques would not rely on the function of endoglin but rather on its high expression on the activated endothelium. Whereas angiogenesis in cardiovascular diseases acts as a disease promoting, inflammation facilitating, and vessel wall weakening process, it may also be harnessed as a therapeutic option to overcome ischemia-related symptoms such as ischemic heart disease or ischemia of muscles in peripheral artery disease [144,145,146,147]. In both cases, imaging of angiogenesis would enable accurate monitoring of disease in a clinical as well as preclinical setting. In the context of abdominal aortic aneurysms (AAAs), current decision-making regarding treatment is based on a balance between patient characteristics, invasiveness of the procedure, and risk of rupture upon a certain aortic diameter (female: >5.0 cm, male: >5.5 cm) [114]. In that regard, noninvasive, endoglin-based PET imaging of intra-aortic angiogenic processes could be of added value in tailoring this decision to a patient-specific level, by possibly identifying patients at increased levels of rupture risk [148,149]. Another example would be endoglin-based imaging of carotid artery plaques to assess lesion stability, as it is known that unstable lesions have a high risk of embolization, which may result in transient ischemic attacks and strokes. If asymptomatic but unstable plaques could be identified via the quantification of angiogenesis, a preventive surgical endarterectomy could be advocated. In case of peripheral artery disease patients, angiogenesis is of paramount importance for treatment and follow-up. Angiogenic wound healing mechanisms, effects of supervised walking therapy, surgical reconstruction, and novel medicinal therapies can potentially be imaged and quantified to predict clinical effects. In the past decade, multiple PET-, MR- and NIRF-based tracers have been developed, but still need improvement to achieve (pre) clinical success (reviewed in [134]). The endoglin-targeting chimeric antibody TRC105 could therefore serve as a non-immunogenic, rapid, and effective method to implement preclinically developed, sophisticated imaging techniques in the clinic.

#### 3.3.2. Endoglin-Based Nuclear Imaging of Cardiovascular Diseases

^64^Cu-NOTA-TRC105-(Fab) has been employed in three different cardiovascular disease models to achieve endoglin-based PET imaging: murine hindlimb ischemia, rat myocardial infarction (MI), mouse calcium-phosphate-induced AAA. In the murine hindlimb ischemia model, the right femoral artery was ligated and excised to mimic peripheral artery disease and image disease-related angiogenesis based on endoglin expression (Table 2) [150]. Gradually decreasing, ischemic hindlimb-specific signal in time (t = 3, 10, 24 days; 48h post-injection) was reported as compared to the contralateral control limb upon PET imaging of the injected tracer in vivo. The highest uptake of ^64^Cu-NOTA-TRC105 was found in the ischemic hindlimb at days 3 and 10, coinciding with near-full compensatory angiogenic mechanisms, as was shown by laser doppler imaging (Figure 3B). Although promising, the results also demonstrated that the relatively long half-life of the tracer caused some background signal in the circulation at 4 h post injection [150]. In a comparable study, it was shown that the ^64^Cu-NOTA-TRC105 tracer could be effectively employed to monitor therapeutic effects of the cholesterol-lowering drug pravastatin in the ischemic hindlimb mouse model [151]. This result, together with its highly specific expression on angiogenic vasculature, make endoglin a promising tracer for monitoring of both progression and treatment of peripheral artery disease. The ^64^Cu-NOTA-TRC105 tracer was also studied in the context of neovessel formation after MI (Figure 3C) [152]. ^64^Cu‑NOTA-TRC105 was injected intravenously in rats that underwent ligation of their left anterior descending artery. PET imaging enabled quantification of neovessel density in the ischemic zone, demonstrating significantly higher signal in the rats that received the MI-inducing surgery compared to sham-operated rats 3 days post-surgery. Tracer uptake remained higher in the MI group on days 10 and 17 post-operation, albeit not significantly, and was confirmed via CD105 immunohistochemistry. Comparable to the hindlimb ischemia model, at 4 and 24 h post-injection, high background signal resulting from the blood impaired effective imaging, while at 48 h, clear visualization of post-MI angiogenesis was possible. An additional signal was observed from the incision in the intercostal muscles, most likely related to angiogenic wound healing processes (Figure 3C) [152]. In the calcium phosphate-induced AAA mouse model, a Fab fragment of TRC105 conjugated with ^64^Cu was used to evaluate angiogenesis in a calcium phosphate-induced AAA mouse model (Table 2) [153]. The advantage of the Fab fragment compared to an intact antibody was, according to ex vivo biodistribution studies, reduced clearance and minimal uptake by the intestines, resulting in reduced background noise. In vivo, the AAA was effectively targeted by ^64^Cu-NOTA-TRC105-Fab and corresponding tracer uptake correlated to immunofluorescent stainings for CD105 [153].

Regarding MR and NIRF imaging for endoglin-based cardiovascular applications, similar results to those above could be achieved in the future, especially when considering the developments in the oncologic research field such as the use of dual-imaging. An interesting application for ultrasound would be to monitor vessel wall stabilization and remodeling upon therapy (e.g., statins). Ultimately, while the optimal choice of tracer (antibody, Fab fragment, nanoparticle) and imaging system (PET, MRI, NIRF, ultrasound) requires additional research, it is clear that endoglin has potential as a target for visualization of cardiovascular diseases.

## 4. Discussion

### 4.1. Summary of Evidence

Based on predominantly preclinical data, endoglin-based imaging shows promising results in both cancer and cardiovascular diseases. PET and MR imaging offer (preoperatively) high-resolution, whole-body, three-dimensional in vivo mapping of lesions, while NIRF imaging has the benefit of real-time visualization, allowing for IGS. CEUS is especially interesting due to the manageability of the appliance in combination with the relatively fast acquisition times and appropriate resolution. The results from the literature search clearly demonstrate that there is a widespread preference for endoglin-based PET imaging. The most commonly researched in vivo model is that of (4T1) breast cancer bearing mice, but the general feasibility of endoglin-based imaging has been demonstrated in many other cancer models, including melanoma, glioma, pancreatic cancer, teratoma, hepatocellular carcinoma, osteosarcoma, and cholangiocarcinoma. Consistent with the preclinical status of the research field, only one paper described endoglin-based imaging in ex vivo human organs, showing effective visualization of renal cell carcinoma vessels in excised human kidneys of patients. As a target of predominantly the neovasculature in cancer, endoglin would be most suited to both (i) serve as a diagnostic and disease monitoring imaging target, to improve current measures of MVD and improve existing diagnostic imaging techniques, (ii) and to function in image-guided surgery, with a potential future to assist surgeons in resecting tumors based on imaging of tumor stroma and neovessels. Neovascularization also plays a central role in the development of cardiovascular diseases and could therefore be harnessed to monitor the development of these pathologies. Although the numbers are limited, the existing studies support the potential of endoglin-based (PET) imaging of angiogenesis in atherosclerosis, in aortic aneurysm, and after myocardial infarction.

### 4.2. Strengths and Limitations

The strength of this review is that it combines all existing studies on endoglin-based imaging, providing a complete overview on (pre) clinically relevant imaging modalities in various in vivo, in vitro, and ex vivo models. The limitation is the heterogeneity of the included studies and its encompassing incomparable outcome measures, confining the report of the results to a narrative synthesis approach.

## 5. Conclusions and Future Perspectives

Research in the field of endoglin-based imaging has mainly been performed by using PET. The diagnostic nature of this modality suggests that there exists either a preference for the development of diagnostic techniques or a lack of focus on endoglin for intraoperative use. Although present in fewer numbers, other endoglin-based imaging techniques such as MRI, NIRF, and ultrasound show effective visualization of the angiogenic endothelium. In the future, multimodal tracers that may be visualized by different imaging techniques hold great promise. Specifically, TRC105 offers the potential to cover the entire care pathway, by using this vector molecule in conjunction with different labels, according to the desired imaging purposes. A notable research field, which has not yet been evaluated in the context of endoglin, is photoacoustic imaging. By exciting tissue with a strong laser of various wavelengths, specific constituents emit sound waves that can be separately captured by an ultrasound transducer. Molecular agents, including antibodies used for NIRF, can emit sound waves upon excitation with these lasers in the NIRF range (700–900 nm), allowing for enhanced contrast. It is evident that a combination of these techniques is of great use in both a preclinical and clinical setting, by combining real-time structural ultrasound visualization and pathophysiological quantification via a compact and manageable system.

The chimeric endoglin-targeting antibody TRC105 seems an ideal candidate to harness the potential of endoglin-based imaging. Therefore, it is unfortunate that the developer of TRC105, TRACON Pharmaceuticals, appears to have lost interest in the antibody’s therapeutic capabilities [154]. As a result, nowadays only a limited number of clinical studies are being performed [155], whereas, based on endoglin’s dynamic potential, this should perhaps be extended to the field of imaging. The most important factor for the latter to succeed is effective translational research. Regardless of the intended tracer, either antibody, peptide or else, endoglin has proven itself as a valuable target for the imaging of cancer and cardiovascular diseases.

## Figures and Tables

**Table 2:** Overview of current endoglin-based cardiovascular disease imaging studies, categorized by imaging modality, disease model, and imaging agent.

Imaging Principle	Specific	Model	Tracer	Class
Nuclear imaging (*n* = 4)	PET (*n* = 4)	Murine hindlimb ischemia	^64^Cu-NOTA-TRC105 [150,151] ^a^	a. Antibody b. Fab fragment
		Rat myocardial infarction (LAD ligation)	^64^Cu-NOTA-TRC105 [152] ^a^	
		Abdominal aortic aneurysm mouse model (calcium phosphate-induced)	^64^Cu-NOTA-TRC105-Fab [153] ^b^	

## Abbreviations

AAA	Abdominal aortic aneurysm
ALK1/5	Activin receptor-like kinase 1/5
AuNP	Gold nanoparticle
BMP	Bone morphogenetic protein
bMSN	Biodegradable mesoporous silica nanoparticle
CEUS	Contrast-enhanced ultrasound
CMCS	Carboxymethyl chitosan
CT	Computed tomography
^64^Cu	Copper-64 radioisotope
CuS	Copper sulfide
dTE	Differential targeted enhancement
DOTA	1:4:7,10-tetraazacyclododecane-1,4,7,10-tetraacetic acid
EPR	Enhanced permeability and retention
^18^F	Fluorine-18 radioisotope
FAP	Fibroblast activation protein
Fe_2_O_3_	Maghemite
^18^F-FDG	18F-Fluorodeoxyglucose
FITC	Fluorescein-5-isothiocyanate
^66^Ga	Gallium-66 radioisotope
Gd-DTPA	Gadolinium-diethylenetriamine pentaacetic acid
HHT1	Hereditary hemorrhagic telangiectasia type 1
hMSN	Hollow mesoporous silica nanoparticle
^125^I	Iodine-125 radioisotope
IGS	Image-guided surgery
iMRI	Intraoperative magnetic resonance imaging
HUVEC	Human umbilical vein endothelial cell
LAD	Left anterior descending
mAb	Monoclonal antibody
MI	Myocardial infarction
mEND	Magnetic endoglin aptamer
Mn_3_O_4_	Manganese oxide
MR(I)	Magnetic resonance (imaging)
mSiO_2_	Mesoporous silica
MSN	Mesoporous silica nanoparticle
MVD	Microvessel density
NIR(F)	Near-infrared (fluorescence)
NOTA	1,4,7-triazacyclononane-1,4,7-triacetic acid
PAMAM	Poly(amidoamine)
PEG	Polyethylene glycol
PESDA	Perfluorocarbon-exposed dextrose albumin
PET	Positron emission tomography
PHEMA	Poly(2-hydroxyethyl methacrylate)
PLA	Poly(l-lactide)
PPAA	Plasma-polymerized allylamine
PVP	Polyvinylpyrrolidone
RGO	Reduced graphene oxide
R-SMAD	Receptor-regulated SMAD
QD	Quantum dot
SPECT	Single photon emission computed tomography
SPION	Superparamagnetic iron oxide nanoparticle
SWCNT	Single-walled carbon nanotube
^99m^Tc	Technetium-99m radioisotope
TGF-β	Transforming growth factor-β
TβRI/II	Transforming growth factor-β receptor I/II
201Tl	Thallium-201 radioisotope
UCNP	Upconversion nanoparticle
VEGF(R)	Vascular endothelial growth factor (receptor)
^86/90^Y	Yttrium-86/90 radioisotopes
^89^Zr	Zirconium-89 radioisotope

## Appendix A. Search Strategy

### Appendix A.1. Component 1: Endoglin

(“Endoglin”[Mesh] OR “Endoglin”[tiab] OR “CD105”[tiab] OR “FLJ41744”[tiab] OR “ORW”[tiab] OR “ORW1”[tiab])

### Appendix A.2. Component 2: Imaging and Image-Guided Surgery

(“Image Guided Surgery”[tiab] OR “Surgery, Image-Guided”[tiab] OR “Image-Guided Surgeries”[tiab] OR “Surgeries, Image-Guided”[tiab] OR “Surgery, Image Guided”[tiab] OR “Image-Guided Surgery”[tiab] OR “Diagnostic Imaging”[Mesh] OR “CT”[tiab] OR “Computed Tomography”[tiab] OR “PET”[tiab] OR “Positron Emission Tomography”[tiab] OR “SPECT”[tiab] OR “Single Photon Emission Computed Tomography”[tiab] OR “Near-infrared”[tiab] OR “Near infrared”[tiab] OR “NIRF”[tiab] OR “Ultrasonography”[tiab] OR “Ultrasound imaging”[tiab] OR “Imaging, ultrasound”[tiab] OR “Ultrasonic imaging”[tiab] OR “Imaging, ultrasonic”[tiab] OR “Ultrasonographic imaging”[tiab] OR “Imaging, ultrasonographic”[tiab])

### Appendix A.3. Combined, Final Strategy

Date of search: 17 December 2020

References found: 476

Combined PubMed search strategy:

((“Endoglin”[Mesh] OR “Endoglin”[tiab] OR “CD105”[tiab] OR “FLJ41744”[tiab] OR “ORW”[tiab] OR “ORW1”[tiab]) AND (“Image Guided Surgery”[tiab] OR “Surgery, Image-Guided”[tiab] OR “Image-Guided Surgeries”[tiab] OR “Surgeries, Image-Guided”[tiab] OR “Surgery, Image Guided”[tiab] OR “Image-Guided Surgery”[tiab] OR “Diagnostic Imaging”[Mesh] OR “CT”[tiab] OR “Computed Tomography”[tiab] OR “PET”[tiab] OR “Positron Emission Tomography”[tiab] OR “SPECT”[tiab] OR “Single Photon Emission Computed Tomography”[tiab] OR “Near-infrared”[tiab] OR “Near infrared”[tiab] OR “NIRF”[tiab] OR “Ultrasonography”[tiab] OR “Ultrasound imaging”[tiab] OR “Imaging, ultrasound”[tiab] OR “Ultrasonic imaging”[tiab] OR “Imaging, ultrasonic”[tiab] OR “Ultrasonographic imaging”[tiab] OR “Imaging, ultrasonographic”[tiab]))
